# Drug induced gingival enlargement - phenytoin: an overview and case report

**DOI:** 10.1093/jscr/rjae304

**Published:** 2024-05-28

**Authors:** Nipun Dhalla, Lipika Gopal, Pooja Palwankar

**Affiliations:** Department of Periodontology, Manav Rachna Dental College, SDS, MRIIRS, Q Block, Faridabad 121001, Haryana, India; Department of Periodontology, Manav Rachna Dental College, SDS, MRIIRS, Q Block, Faridabad 121001, Haryana, India; Department of Periodontology, Manav Rachna Dental College, SDS, MRIIRS, Q Block, Faridabad 121001, Haryana, India

**Keywords:** gingival enlargement, epilepsy, phenytoin, periodontal disease

## Abstract

Gingival enlargement is a side effect of several different medication, including immunosuppressants, anticonvulsants, and calcium channel blockers. It is an inflammatory response that starts when plaque and calculus build up on the tooth surface. The most prevalent long-term neurological condition affecting people is epilepsy. In affluent nations, the prevalence of epilepsy is ~ 1%, whereas in less developed countries, it may >2%. The preferred medication for the condition, phenytoin, has major side effects include gingival enlargement. In addition to being visually disfiguring, this enlargement frequently affects speech, chewing and eating. Furthermore, those with poor dental hygiene, causes disabilities with motor coordination and muscular limitations leading to mental disability and physical impairments are more prone to periodontal disease. This article enlightened the mechanism of drug induced gingival enlargement clinically, microbiologically, and surgically.

## Introduction

Gingival enlargement is an enlargement of the gingival tissues that can be localized or generalized. Gingival hyperplasia (increase in cell number) and gingival hypertrophy (increase in cell size) are histological diagnoses that do not adequately describe the pathological processes seen inside the tissues [1]. Various factors such as adverse drug effects, inflammation, neoplastic processes, and hereditary gingival fibromatosis have been linked with enlargement [2].

Drug-induced gingival enlargement is a side-effect of the administration of drugs where the gingival tissue is not the intended target organ [3].

They are classified as anticonvulsants (phenytoin [PHT], valproate, carbamazepine), immunosuppressants (cyclosporine, tacrolimus), and calcium channel blockers (nifedipine, verapamil). Among the drugs, PHT shows enlargement in up to 50% of patients who take this drug, as this has been widely related to treating epilepsy as an antiepileptic drug [4].

The most prevalent chronic neurological condition in humans is epilepsy. Epilepsy affects ~1% of the population in affluent countries, and it affects 2% of the population in less developed countries [5]. Drug-based therapies that help patients become seizure-free are the foundation of epilepsy treatment [6].

Merritt and Putnam (1937) [7], first, introduced an anticonvulsant or antiepileptic drug, known as PHT. The first enlargement case associated with the chronic use of PHT was in 1939 [8].

The first line of treatment is a non-surgical one, which includes adequate plaque control and discontinuing or altering the stimulant drug. Only when medical treatment is unsuccessful, surgical management become necessary, however, recurrences are common and the effects typically endure for 12 months [9].

### Clinical and microscopic features

The onset of growth starts within first month, but the occurrence rate is within 3 months and reaches the peak within first year of the medication [10].

Clinically, it begins in the interdental papillae, which increases and forms a mass affecting the greatest in the buccal surfaces of the anterior region (maxillary and mandibular both) and may cover the whole tooth crown [8].

Microscopically, there is an excessive collection of extracellular matrix protein along with rete pegs penetrating the underlying connective tissue. A large number of infiltrated inflammatory cells are seen, mostly plasma cells [11].

### Pathogenesis and mechanism

Many factors interact to cause gingival overgrowth to develop. PHT restricts the production of extracellular matrix and lowers the activity of collagenase, thereby causing a decline in CA2+ cell inflow, which inhibits folic acid absorption. The drug decreases collagen endocytosis by causing fibroblasts to express α2$ \beta $1-integrins. Myofibroblasts are subsequently activated as a result of lower expression of type I and III collagen mRNA [8].

PHT-induced gingival overgrowth is closely related to cytokines. PHT-stimulated fibroblasts produce substantial amounts of IL-6, IL-1, and IL-8. The immune system and connective tissue are directly connected by these mediators, which can stimulate T cell proliferation and dispatch neutrophils to the tissues. This relationship seems to be closely related to fibrotic diseases [12].

Dental plaque may have contributed to the onset because it causes the production of a local inflammatory response that is critical to the development of gingival enlargement. Growth factors such as connective tissue growth factor (CTGF), platelet derived growth factor, fibroblast growth factor, and transforming growth factor - beta (TGF-$ \beta $) are more prevalent in fibrotic tissues and are associated with PHT enlargement. PHT may influence the production of IL-13 by promoting Th2 cells. Additionally, it might lead to the release of TGF, CTGF, and other growth factors from macrophages, all of which support the fibrotic lesions usual activities such as collagen manufacture, fibroblast proliferation, activation of Tissue inhibitors of metalloproteinases (TIMPs), inhibition of matrix metalloproteinases (MMPs), and extra cellular matrix (ECM) synthesis [13].

## Procedure

A 20-year-old female patient, reported gingival enlargement to the Department of Periodontology, having a chief complaint of halitosis, bleeding gums, and aesthetics for 2 years. Medical history was taken and the patient revealed that she had been taking PHT 100 mg BD since she was 16 years of age as she had an episode of seizures (epilepsy).

**Figure 1:**
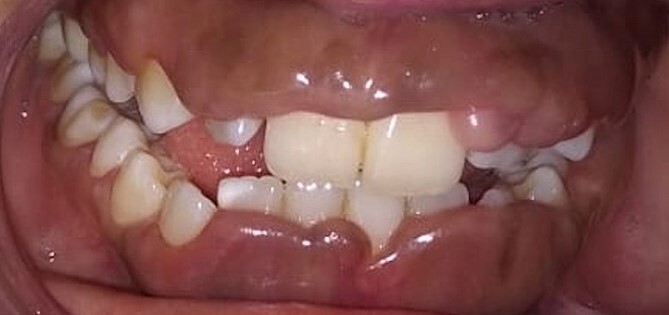
Phenytoin induced gingival enlargement.

Intraoral examination shows generalized puffiness and swollen gingiva, mainly in the upper and lower anterior region covering two-third of the entire teeth, the color of the gingiva was bluish red, which bleeds on slight provocation as well as on mastication of hard food. (Fig. 1).

The patient also gave the history of change of drug 6 months back, following the consent of the physician non-surgical periodontal therapy was performed. Chlorhexidine 0.12% mouthwash and proper brushing technique were demonstrated and recalled after 15 days for further treatment.

Based on medical history a provisional diagnosis of PHT drug-induced gingival enlargement was made.

On recall and review, a surgical intervention, gingivectomy was planned, i.e. surgical excision of gingival tissues.

### Surgical intervention

**Figure 2:**
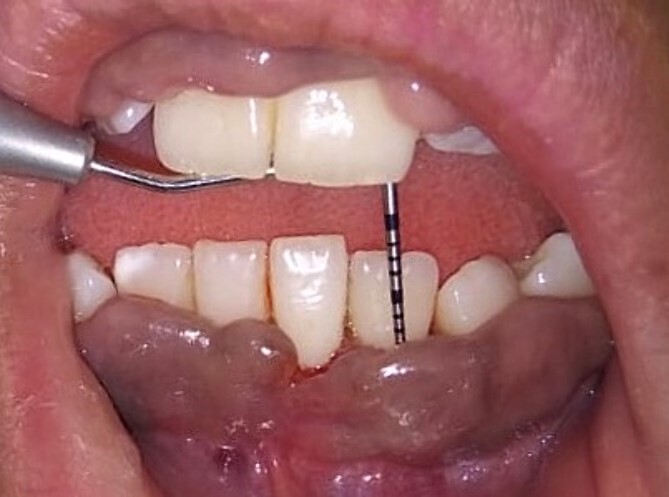
Measuring the periodontal pocket of lower anteriors showing gingival enlargement.

**Figure 3:**
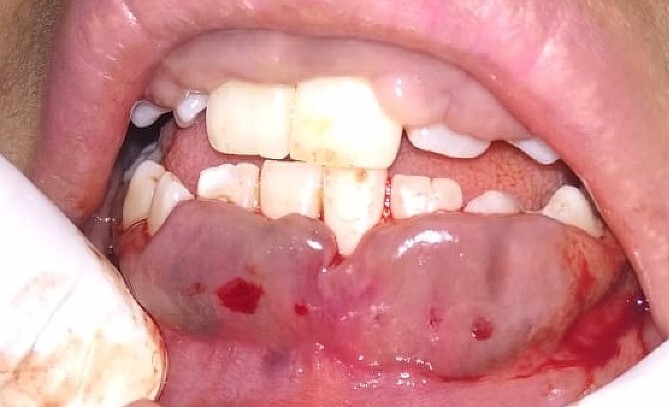
Bleeding points marked.

**Figure 4:**
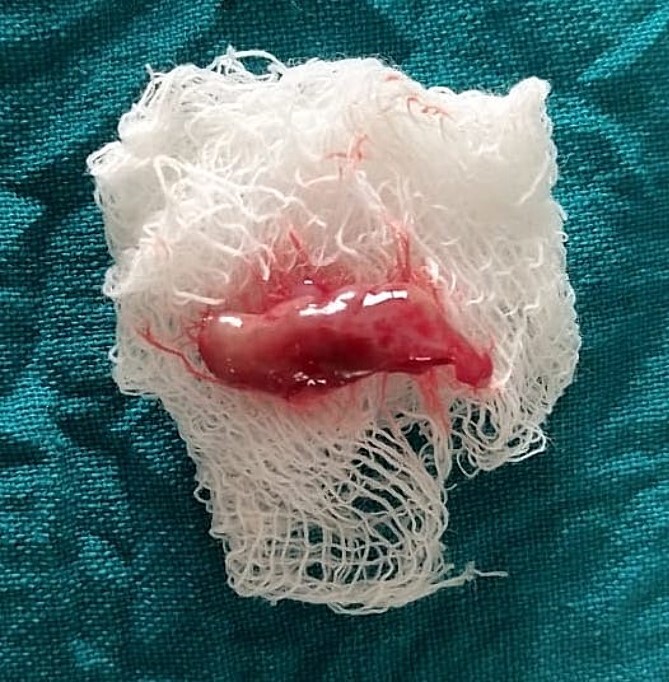
Excised tissue.

**Figure 5:**
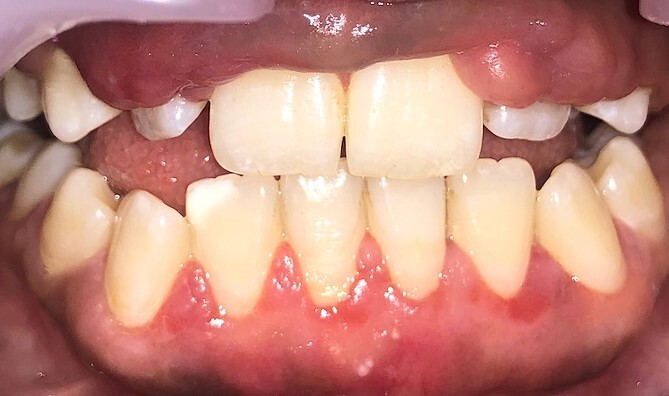
Post healing of gingiva.

On the day of the procedure, a complete blood test was done which shows normal limit values. Following the administration of local anesthesia, periodontal pockets were checked (Fig. 2) and the bleeding points were marked using a Krane Kaplan pocket marker, (Fig. 3) continuous incision was made with the scalpel and blade no. 15, keeping the bevel at ~45° to the tooth surface. Once the incisions had been made, the excision of the tissue was done with a curette (Fig. 4). Further electrocautery was used as a coagulating machine to control the bleeding. Gingivoplasty, i.e. reshaping and recontouring of gingiva following the normal festooned pattern was done. Coe-Pak was placed and the patient was recalled after 1 week for check-up (Fig. 5).

### Histopathology

**Figure 6:**
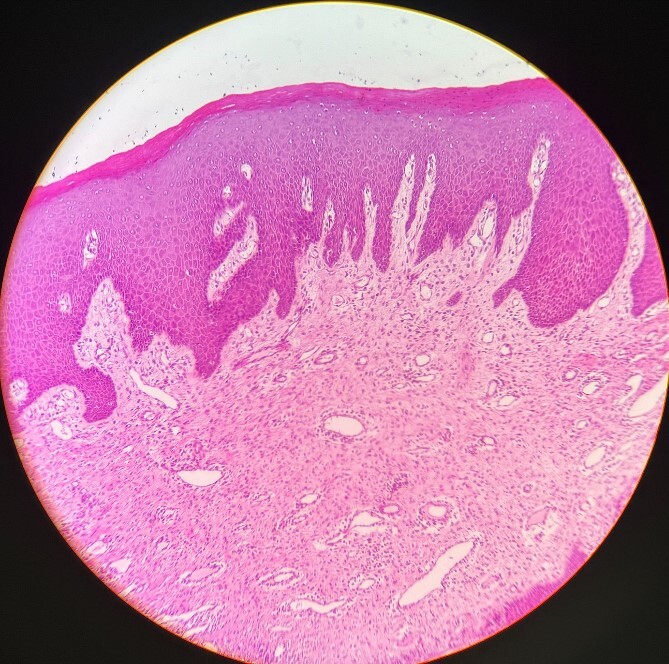
Histopathological picture showing hyperplastic para keratinized stratified squamous epithelium with long rete ridges, chiefly, inflammatory cells in the connective tissue.

The H&E stained tissue section reveals a hyperplastic para keratinized stratified squamous epithelium with long rete ridges. The underlying connective tissue is fibroreticular with dense bundles of collagen fibers showing varying numbers of fibroblast and infiltration of chronic inflammatory cells chiefly, lymphocytes, and plasma cells. Blood vessels of varying size and shape are also evident (Fig. 6).

## Discussion

The administration of anticonvulsants, calcium channel blockers, and immunosuppressants shows drug-induced gingival enlargement as a possible risk factor, and poor oral hygiene is observed to be the attributable factor to it [14].

The research projected that the mechanism of enlargement shows variation in calcium metabolism, metalloproteases, and expression of integrin due to disruption caused by homeostasis of collagen degradation and synthesis. This fibrosis is characterized by the presence of numerous fibroblasts with an activated synthetic and proliferative phenotype that can be influenced by deregulated cytokines [15].

Several factors could influence these gingival alterations such as the kind of drug, its dosage, interactions with other medications, current oral health practices, plaque build-up, other periodontal conditions, and individual response variability of genetic variables influencing heterogeneity of gingival fibroblast [4].

Extensive gingival enlargement can cause both psychological and physical draining. The gingival volume usually increases gradually, but in extreme situations, the gingival tissue may cover all of the teeth, making it difficult to speak and chew. Additionally, gingival enlargement can result in depression and nervousness, particularly if it affects aesthetics and one’s ability to smile and make facial expressions [16].

The treatment often centres on managing local inflammatory mediators such as calculus and plaque effectively and substituting drugs. Resolving the issue may involve stopping the medication that is causing the problem and practicing strict dental hygiene, yet surgical intervention is typically needed [17].

Dental redirection is advised for people taking PHT, not only to alleviate the ailment but also to increase the incentive to maintain proper oral hygiene.

## Data Availability

The data set used in this study is available on request.

## References

[1] Sabarudin M , Taib H, Wan MW. Refining the mechanism of drug-influenced gingival enlargement and its management. Cureus 2022;14:e25008–9. 35712334 10.7759/cureus.25009 PMC9195644

[2] Ramirez RA , Brunet LL, Lahor SE, Miranda RJ. On the cellular and molecular mechanisms of drug-induced gingival overgrowth. Open Dent J 2017;11:420–35. 28868093 10.2174/1874210601711010420 PMC5564016

[3] Marshall RI , Bartold PM. Medication induced gingival overgrowth. Oral Dis 1998;4:130–51. 9680902 10.1111/j.1601-0825.1998.tb00269.x

[4] Urolagin SS , Swaroop D, Agrawal C, Dholakia P, Karalwad MB. Management of phenytoin-induced gingival enlargement in a patient with antiphospholipid antibody syndrome: a rare case report. J Indian Soc Periodontol 2016;20:561–4. 29242694 10.4103/0972-124X.201693 PMC5676340

[5] Guidelines for epidemiologic studies on epilepsy . Comission on epidemiology and prognosis, international league against epilepsy. Epilepsia 1993;34:592–6. 8330566 10.1111/j.1528-1157.1993.tb00433.x

[6] Sander JW , Shorvon SD. Epidemiology of the epilepsies. J Neurol Neurosurg Psychiatr 1996;6:433–43. 10.1136/jnnp.61.5.433 PMC1074036 8965090

[7] Arnold D , Steinberg. Clinical management of phenytoin-induced gingival overgrowth in handicapped children. Pediatr Dent 1981;3:130–6.

[8] Correa JD , Queiroz CM, Costa JE, Teixeira AL, Silva TA. Phenytoin-induced gingival overgrowth: a review of the molecular, immune, and inflammatory features. ISRN Dent 2011;2011:497850. 21991476 10.5402/2011/497850 PMC3168966

[9] Tungare S , Paranjpe AG. Drug-Induced Gingival Overgrowth StatPearls [Internet]. Treasure Island. FL: StatPearls Publishing, 2024,

[10] Lin K , Guilhoto L, Yacubian E. Drug-induced gingival enlargement - part II. Antiepileptic drugs: not only phenytoin is involved. J Epilepsy Clin Neurophysiol 2007;13:83–8.

[11] Drozdzik A , Drozdzik M. Drug-induced gingival overgrowth—molecular aspects of drug actions. Int J Mol Sci 2023;24:5448. 36982523 10.3390/ijms24065448 PMC10052148

[12] Farook FF , Nizam M, Alshammari A. An update on the mechanisms of phenytoin induced gingival overgrowth. Open Dent J 2019;13:430–5.

[13] Nickel J , Ten Dijke P, Mueller TD. TGF-β family co-receptor function and signalling. Acta Biochim Biophys Sin 2018;50:12–36. 29293886 10.1093/abbs/gmx126

[14] Hegde R , Kale R, Jain AS. Cyclosporine and amlodipine induced severe gingival overgrowth - etiopathogenesis and management of a case with electrocautery and carbon-dioxide (CO_2_) laser. J Oral Health Comm Dent 2012;6:34–42.

[15] Kataoka M , Kido J, Shinohara Y, Nagata T. Drug-induced gingival overgrowth—a review. Biol Pharm Bull 2005;10:1817–21. 10.1248/bpb.28.1817 16204928

[16] Estella K , Ana L, Luminitt L. Drug-induced changes in the gingival tissue. J Interdiscip Med 2023;8:1–5.

[17] Chacko LN , Abraham S. Phenytoin-induced gingival enlargement. BMJ Case Rep 2014;2014:bcr2014204670. 10.1136/bcr-2014-204670 PMC4039754 24872495

